# Overall process of using a valerate-dominant sludge hydrolysate to produce high-quality polyhydroxyalkanoates (PHA) in a mixed culture

**DOI:** 10.1038/s41598-017-07154-3

**Published:** 2017-07-31

**Authors:** Jiuxiao Hao, Xiujin Wang, Hui Wang

**Affiliations:** State Key Joint Laboratory of Environment Simulation and Pollution Control, School of Environment, Tsinghua University, Beijing, 100084 China

## Abstract

The overall process of polyhydroxyalkanoates (PHA) production in a mixed culture fed by thermophilic fermented valerate-dominant sludge hydrolysate with high-level soluble organics (proteins and carbohydrates) and nutrients (nitrogen and phosphorus) was investigated in this study. The valerate-dominant hydrolysate was fed to enrich a PHA culture with an increasing concentration, and the enriched culture displayed a strong PHA-producing capacity under feast-famine conditions. Valerate in the feedstock was preferentially utilized over acetate and butyrate, and its uptake correlated with the production of 3-hydroxyvalerate (3HV) and 3-hydroxy-2-methylvalerate (3H2MV). The maximum PHA content (42.31%) was highest to date in a mixed culture with complex feedstock, and the PHA consisted of 3-hydroxybutyrate (3HB), 3HV, 3H2MV at 68.4, 23.7, 7.9 mmol C%. PHA production was inhibited when the nutrients exceeded a certain limit. Microbial analysis revealed that valerate-dominant feedstock caused *Delftia* (53%) to become the prevailing group over other PHA-producing bacteria. For long-term operation, 75% of the biomass at the end of feast phase was collected for PHA recovery, and the entire process exhibited a potential to produce 5 g PHA from 1 kg sludge. These findings indicate that the complex valerate-dominant sludge hydrolysate can be used to stably produce PHA containing high 3HV and 3H2MV.

## Introduction

Polyhydroxyalkanoates (PHA) are biopolyesters synthesized by a wide range of bacteria and can functionally replace the conventional petroleum-based plastics^1^. To reduce the high PHA production costs and the environmental footprint, many efforts have been devoted to transforming PHA production from pure culture with pure substrates to mixed microbial cultures (MMCs) with inexpensive waste substrates^2–4^. The typical process of obtaining PHA from waste includes three stages^5^: first, anaerobic fermentation is adopted to convert the complex organic wastes into volatile fatty acids (VFAs), which are the preferred precursors for PHA synthesis; second, MMCs with a high PHA-storing capacity are enriched from activated sludge or other sources; third, after the enriched MMCs are physiologically conditioned for polymer synthesis, the biomass is harvested to inoculate a batch reactor fed by the fermented VFAs, wherein the quantities of PHA are accumulated and recovered. In practice, the enrichment can be accomplished by subjecting the MMCs to selective pressure caused by consecutive periods of external substrate excess (feast) and limitation (famine) in a sequencing batch reactor (SBR), referred to as a feast-famine regime or aerobic dynamic feeding (ADF)^6^.

One main factor affecting MMC PHA production is the components of the fermented hydrolysate, including VFAs, non-VFAs, and nutrients^3^. The compositions of VFAs in the feedstock, i.e., the relative proportions of acetate, propionate, butyrate and valerate, directly affect the yield, productivity and monomer composition of the produced PHA^7^. It was traditionally considered that the PHA-producing MMCs preferentially used even-carbon VFAs (acetate and butyrate) over odd-carbon VFAs (propionate and valerate), and even-carbon VFAs usually resulted in a higher PHA yield and productivity^8, 9^. However, the effects of VFAs on the PHA production efficiency also rely on the cultivating substrate during enrichment^10^ as well as the microbial community of the enriched culture^11^. In addition, the molecular composition of PHA is dependent on the VFAs species provided^7^. Generally, even-carbon VFAs tend to generate 3-hydroxybutyrate (3HB) monomer, whereas odd-carbon VFAs yield 3-hydroxyvalerate (3HV) and other longer-chain monomers^1^, which has been clearly evidenced by studies employing simple substrates, such as sole VFA or a VFAs mixture^8^. The monomer composition of PHA subsequently determines the thermal and mechanical properties of the polymer, including its elasticity, crystallinity and rigidness^12^. The homopolymer poly(3-hydroxybutyrate) (PHB) is a very crystalline and stiff material with a high melting point, which hinders its processing and commercial applications^13^. To improve the properties of pure PHB, incorporating 3HV and other longer-chain monomers will lead to ameliorative attributes, such as a decreased melting point and increased ductility^14^.

On one hand, regarding the real fermented waste streams, even-carbon VFAs therein usually account for a greater fraction than odd-carbon VFAs^5, 15^, and most researchers have focused on hydrolysates mainly composed of even-carbon VFAs^3, 11^. On the other hand, odd-carbon VFAs may also become the dominant products in the hydrolysates^16, 17^, as the components of fermented streams can be regulated; for instance, the proportions of propionate and valerate were increased by adjusting the type of wastes, inoculum, fermentation conditions, and other parameters^16, 18^. However, to date, the hydrolysates dominated by odd-carbon VFAs have been little studied in the field of MMC PHA production. Bengtsson *et al*. used a propionate-dominant hydrolysate (a fermented paper mill wastewater with 40% propionate) to accumulate PHA in the enriched activated sludge. They observed that acetate was taken up at the highest specific rate, followed by propionate and butyrate at similar rates, with valerate taken up at the slowest rate. The resultant copolymer poly(3-hydroxybutyrate-co-3-hydroxyvalerate) (PHBV) yielded 48 wt% content, consisting of 47 mol% 3HB and 53 mol% 3HV^19^. However, except for this investigation, there has been no study exploiting the valerate-dominant fermented hydrolysate for PHA production, either for enrichment or for synthesis, and valerate has only been reported in studies using sole valerate or a valerate-containing mixture^8, 20, 21^. Recently, valerate-dominant hydrolysates were reported to be produced from the anaerobic fermentation of excess sludge^22, 23^, especially under thermophilic conditions and with protein-rich sludge^17^. Therefore, it is highly necessary to determine whether this type of valerate-dominant hydrolysate is suitable and efficient as a PHA-producing feedstock and what PHA product will be obtained.

In addition to VFAs as the main products, the non-VFA organics (proteins and carbohydrates) and nutrients (nitrogen and phosphorus) are non-negligible in the fermented streams, and they usually exert adverse effects on PHA accumulation^24^. The non-VFAs were consumed more slowly than VFAs^10^, and they contributed mostly to growth but not PHA synthesis^25^, leading to lower PHA contents than when using pure-VFAs substrates^9^. Furthermore, the presence of non-VFAs would increase the survival of non-PHA-producing bacteria and impair the PHA synthetic ability of the enriched MMC^10^. The levels of nitrogen (N) and phosphorus (P) are recognized as being pivotal to balancing PHA storage and microbial growth^26^. In general, the PHA production process preferred nutrient-limited conditions to minimize biomass growth and maximize the stored PHA^27^. The sludge fermented hydrolysate is a typical VFAs-rich renewed stream that contains high levels of non-VFAs, N and P and has been widely applied to PHA production^3, 9^. Most existing studies removed N and P from the hydrolysate before use^28, 29^; moreover, a supernatant discharge step was adopted immediately after the feast phase to eliminate the non-VFAs^25^. These operations inevitably increased the complexity and production costs of the overall process. Although few studies used the raw fermented streams with non-VFAs and excess N and P for PHA production^24, 30^, the feedstocks in these studies were all characterized by dominant acetate, and the accumulated PHA contents were relatively low. In this respect, the direct utilization of valerate-dominant sludge fermented hydrolysate remains unknown.

The aim of the present work was to evaluate the feasibility and potential of using a valerate-dominant hydrolysate for PHA production by the enriched MMC. The hydrolysate was from sludge thermophilic fermentation and inherently involved high levels of non-VFAs and nutrients. In conjunction, the relationships between substrate utilization (including VFAs, non-VFAs and nutrients), microbial growth, and PHA synthesis, as well as the microbial composition of the enriched MMC, were studied to investigate the PHA synthetic characteristics of the overall process and to reveal the effects of the valerate-dominant hydrolysate as feedstock on PHA production.

## Results

### Enrichment of the PHA-producing mixed culture by the valerate-dominant sludge hydrolysate

For the VFAs production in the batch sludge reactor during 5-day anaerobic fermentation, the maximal SCOD and VFAs concentrations both occurred at 60 h (Fig. S1a). The ratio of VFAs to SCOD reached 61.55% at 12 h, increased to the highest value of 71.32% at 60 h, then began to decrease. The parameter degree of acidification (DA) represented the acidification efficiency of the fermentation process^31^, and the highest DA (35.17%) also occurred at 60 h. Therefore, 60 h was the optimal time to collect the fermented hydrolysate for further use. The characteristics of the collected hydrolysate are shown in Table 1. Acetate, butyrate and valerate were the main products, especially valerate, accounting for more than 50% of the total VFAs (COD basis), while the amount of propionate was quite small. The hydrolysate was directly used for the subsequent culture enrichment and PHA accumulation without any component removal or other disposals.

**Table 1:** Characteristics of the sludge thermophilic fermented hydrolysate collected at 60 h.

Parameter	Sludge fermented hydrolysate
SCOD (mg/L)	8243 ± 309
Acetate (mg COD/L)	1184 ± 20 (20.14%)
Propionate (mg COD/L)	138 ± 6 (2.35%)
Butyrate (mg COD/L)	1496 ± 41 (25.46%)
Valerate (mg COD/L)	3060 ± 72 (52.05%)
soluble protein (mg COD/L)	665.9 ± 10.3
soluble carbohydrate (mg COD/L)	277.7 ± 18.4
NH_4_-N (mg/L)	475.28 ± 26.21
PO_3_-P (mg/L)	45.87 ± 1.38
pH	6.87 ± 0.36

The results are the averages and their deviations for different batches (≥6). The percentages in parentheses after the values of acetate, propionate, butyrate and valerate are on a COD basis in total VFAs.

Considering that the total organic concentration (SCOD) and VFAs were very high in the hydrolysate and that valerate was more difficult to be used than shorter-chain acids such as acetate and propionate^20, 32^, a special enrichment method with increasing initial concentrations was adopted to improve the adaptability and stability of the MMC. The entire enrichment process lasted for approximately 200 days (Fig. S2). Table 2 demonstrates the primary parameters for each concentration during the enrichment. After 75 cycles, the MMC was well adapted to the final undiluted hydrolysate concentration. The time needed to consume the supplied COD increased along the cycles, but the actual substrate utilization rate increased rapidly, especially at the later stage of enrichment. The maximum PHA content gradually improved and ultimately reached over 40%, which was 8 times higher than that of the first cycles with an SCOD of 500 mg/L.

**Table 2:** The primary parameters for the whole process of enriching PHA-producing MMC fed by valerate-dominant sludge hydrolysate with increasing initial concentrations.

Cycle	SCOD (mg/L)	Feast phase (h)	CDW (g/L)	Maximum PHA content (%)	PHA concentration (g/L)
1~7	500	9	0.92	5.00	0.05
8~16	1000	12	1.66	8.74	0.15
17~26	2000	18	2.02	16.70	0.34
27~35	3000	18	2.30	23.94	0.55
36~47	4000	21	2.96	33.69	1.00
48~60	6000	24	2.88	38.69	1.11
61~75	8243	24	3.08	42.34	1.30

The results of ‘feast phase’, ‘CDW’, ‘maximum PHA content’ and ‘PHA concentration’ are the average of the last three batches for each concentration. ‘CDW’ and ‘PHA concentration’ indicate the values at the end of feast phase.

In most previous studies, to fully exert the PHA-storing capacity of the enriched MMCs and achieve the highest PHA content, the biomass at the end of the famine phase was transferred to batch reactors as a separate accumulation stage, where a pulse-wise feeding strategy and a nutrient-absent condition were carried out^4, 10^. By comparison, the enriched MMC in the present study already functioned properly to synthesize PHA during the feast-famine operation and reached a comparable PHA content. Therefore, the separate PHA accumulation stage could be omitted, and the conventional three-stage MMC PHA production process was simplified to two stages: (1) anaerobic fermentation to obtain a VFAs-rich substrate and (2) cyclic PHA production and recovery after the MMC was well enriched (Fig. S3). To continuously obtain PHA products, in the second stage at the end of the feast phase, one portion of the biomass was withdrawn for polymer recovery and the other portion was left in the SBR for the next new cycle (details in Fig. 5).

### Utilization of VFAs, non-VFAs and nutrients in the feedstock during one cycle

**Figure 1:**
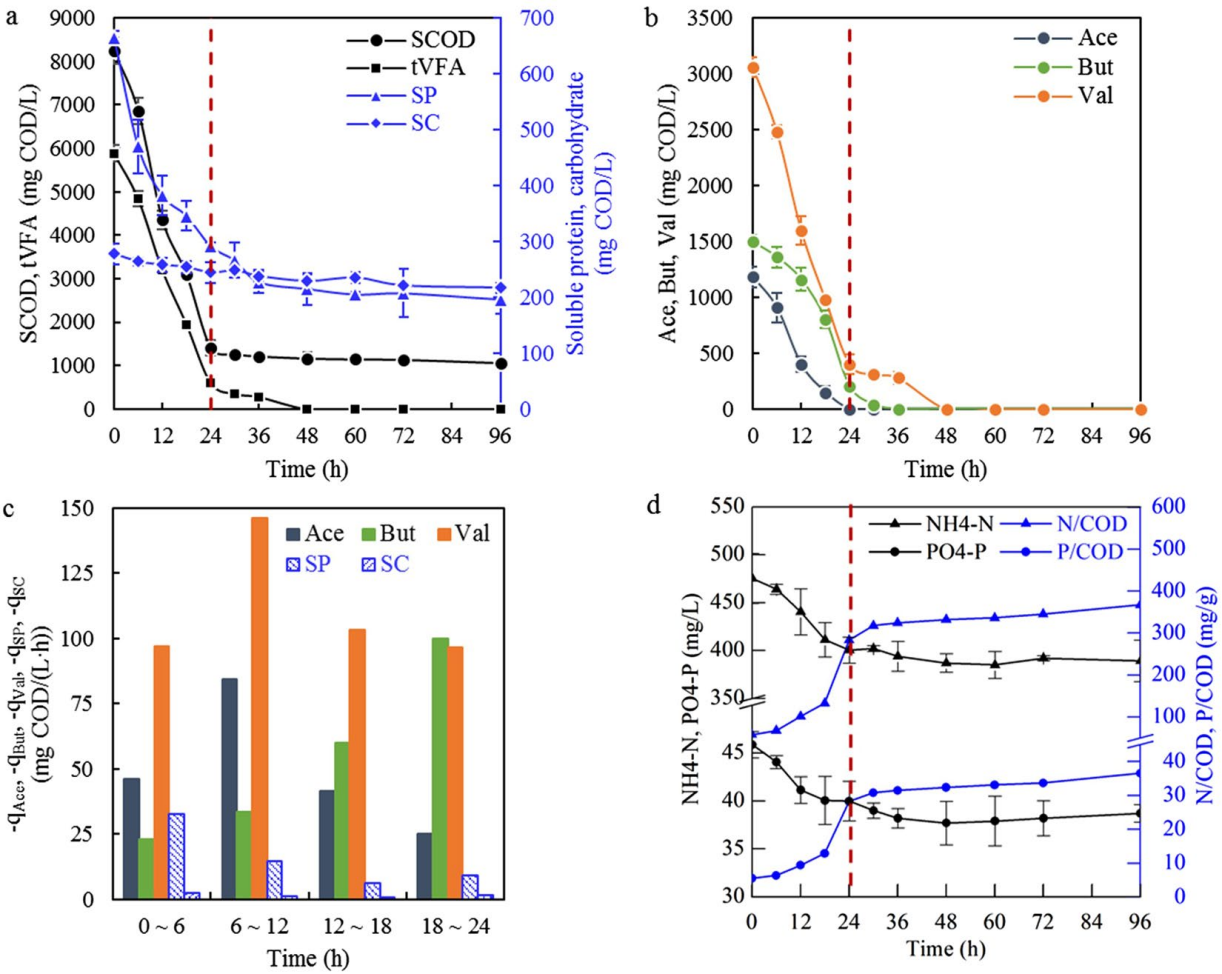
Profiles of SCOD, total VFAs, soluble proteins and carbohydrates (**a**), main VFAs (**b**), their uptake rates (**c**), and nutrients utilization (**d**) during one cycle in the SBR operated under stability. Red dashed lines mark the end of feast phase. The uptake rate represents the average for the marked period. ‘tVFA’: total VFAs, ‘Ace’: acetate, ‘But’: butyrate, ‘Val’: valerate, ‘SP’: soluble proteins, ‘SC’: soluble carbohydrates. (bars = S.D., n = 3).

Figure 1 shows the cyclic concentrations of different components in the feedstock and the uptake rates of VFAs and non-VFAs under stable operation. Upon the commencement of substrate addition for a new cycle, more than 80% of the SCOD was consumed within the first 24 h, and the residual (approximately 1000 mg/L) was recalcitrant to be removed even in the famine phase (Fig. 1a). The remaining substrate mainly consisted of soluble protein, soluble carbohydrate and inert substances. The VFAs were quickly absorbed and assimilated for both bacterial growth and PHA synthesis during the feast phase, and the remainder passed into the famine phase, being used up at 48 h. Among the three main VFAs in the hydrolysate (Fig. 1b,c), valerate was consumed much faster than acetate and butyrate. Before 12 h, the MMC chiefly utilized acetate and valerate, and their uptake rates increased during this period. Although the initial amount of butyrate surpassed that of acetate (Table 1), its consumption by the MMC fell behind for the earlier feast phase. For the latter feast phase from 12 h to 24 h, the uptake rates of acetate and valerate decelerated, while that of butyrate distinctly accelerated; in particular, between 18 h and 24 h, valerate and butyrate had similar utilization rates. The average consumption rates of acetate, butyrate and valerate in the feast phase were 49.3, 54.0, and 110.8 mg COD/(L·h), respectively.

Soluble proteins and carbohydrates were the major non-VFAs in the fermented hydrolysate, and initially, they respectively accounted for 8.08% and 3.37% in the total SCOD (Table 1). As shown in Fig. 1a, the MMC showed a greater demand for proteins than carbohydrates; the cyclic uptake ratio of proteins was 70.57%, while that of carbohydrates was only 21.86%. In contrast to VFAs, the uptake rates of non-VFAs were obviously slower. The average uptake rates of the total VFAs and total non-VFAs (the sum of proteins and carbohydrates) in the feast phase were 219.96 and 17.00 mg COD/(L·h), respectively. The consumption of N and P occurred simultaneously with the absorption of carbon sources in the feast phase and continued in the famine phase until 48 h (Fig. 1d). As the carbon sources were consumed at higher rates than nutrients, N/COD and P/COD increased throughout, especially during the feast phase.

### PHA synthesis and biomass growth of the enriched culture

**Figure 2:**
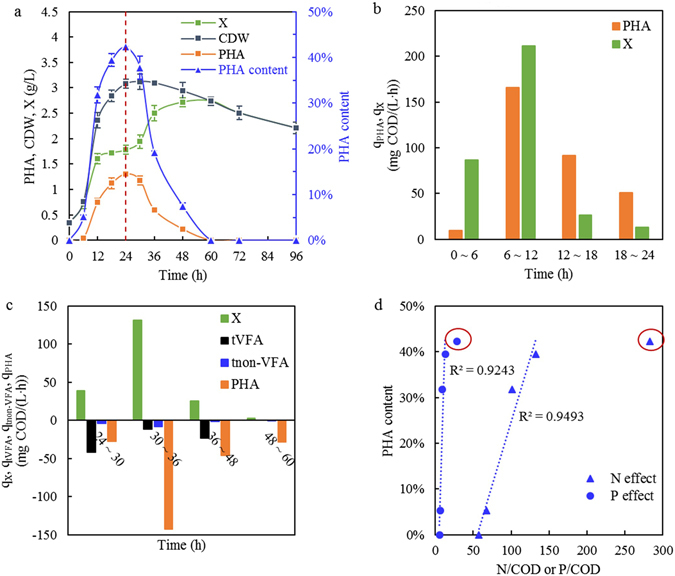
Profiles of PHA and biomass concentration during one cycle (**a**), the specific production rates in feast phase (**b**), the degradation and utilization rates in famine phase (negative values) (**c**), and the effects of nutrients on PHA content (**d**) in the SBR operated under stability. Red dashed lines mark the end of feast phase. Red circles mark the outliers away from the trend lines. Figure d only shows the data in feast phase. (bars = S.D., n = 3).

The profiles of PHA synthesis, degradation, and biomass growth in one cycle are illustrated in Fig. 2. The stored PHA and biomass (X) increased simultaneously in the feast phase, and the maximum PHA concentration (1.30 g/L) occurred at the end of the feast phase, when X reached 1.78 g/L and CDW reached 3.08 g/L (Fig. 2a). At the onset of a new cycle, the MMC had to replenish some necessary intracellular compounds after the prior starvation; thus, the growth exceeded the PHA synthesis and then, after 12 h, PHA production gradually overcame the growth (Fig. 2b). From 12 h to 24 h, X changed slowly, implying that almost all substrates consumed within this period were oriented to the PHA polymer. The change in PHA content was similar to that of the PHA concentration, and the largest value was 42.31% at the end of the feast phase. Due to the carbon transfer from PHA to biomass in the famine phase, CDW decreased much more slowly than PHA, and thus, the PHA content decreased quickly after 24 h. The carbon source for biomass growth in the famine phase derived from three factors: the residue VFAs, the non-VFAs and the stored PHA (Fig. 2c). Comparatively, PHA were the primary source, except between 24 h and 30 h, when they were overtaken by the residue VFAs, and the non-VFAs contributed much less. As shown in Fig. 2d, the PHA content increased with an increase in the ratios N/COD and P/COD, and they had close correlations. However, when N/COD exceeded 150 mg/g and P/COD exceeded 15 mg/g, PHA production was severely inhibited.

**Figure 3:**
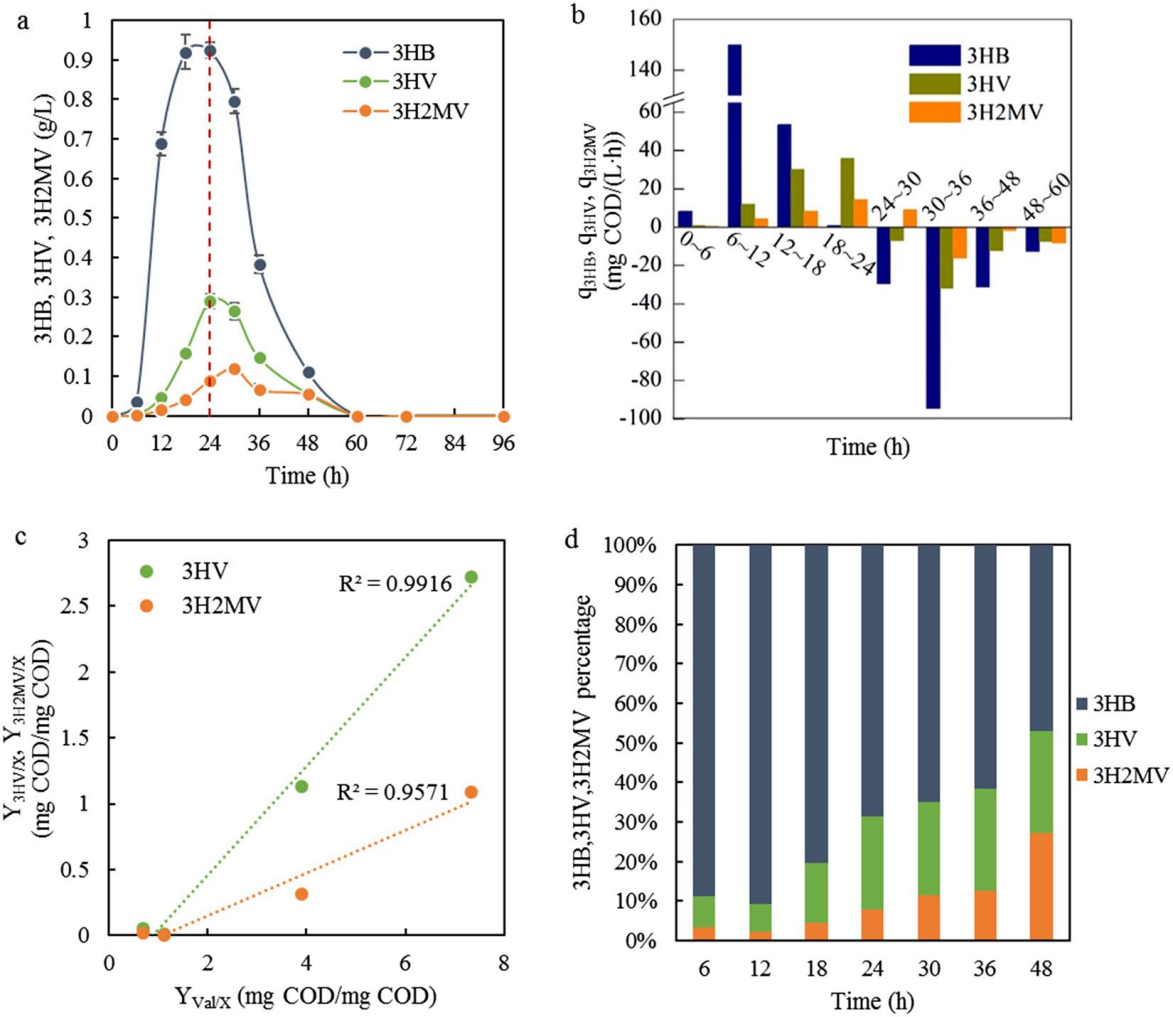
Profiles of 3HB, 3HV and 3H2MV monomers (**a**), their production and degradation rates (negative values) (**b**), the effects of valerate accessibility on 3HV and 3H2MV production in feast phase (**c**), and the monomers proportion (**d**) during one cycle in the SBR operated under stability. Red dashed lines mark the end of feast phase. The percentages of 3HB, 3HV and 3H2MV are on molar carbon basis. (bars = S.D., n = 3).

The produced PHA polymer was composed of 3HB, 3HV and 3-hydroxy-2-methylvalerate (3H2MV). 3H2MV belongs to mcl-PHA, and it imparts more beneficial characteristics to PHA than 3HV, as it has two branches at both α- and ß-carbons, making the polymer more resistant to lysis and decomposition^33^. The synthesis of 3HB mainly occurred between 6 h and 18 h (Fig. 3a,b), and the production rate reached up to 149.97 mg COD/(L·h) for the period 6 h to 12 h. The synthesis of 3HV and 3H2MV started from 6 h, and the rates escalated along the feast phase. At the later feast phase (18 h to 24 h), there was almost no 3HB produced, and only 3HV and 3H2MV were synthesized. The amount of propionate in the sludge fermented hydrolysate was negligible (Table 1), and therefore, the production of 3HV and 3H2MV was significantly attributable to valerate.

Figure 3c shows that, during the feast phase, valerate uptake per biomass was highly linearly correlated with the production of 3HV and 3H2MV. The stored 3HB and 3HV began to be utilized entering the famine phase, but 3H2MV could still be generated until 30 h, probably because of the few remaining VFAs in the feedstock. 3HB was first produced in the feast phase and also first degraded in the famine phase; by contrast, the other two longer-chain monomers were produced and degraded more slowly. At the later PHA degradation stage (48 to 60 h), the degradation rates of all three monomers tended to be similar. In regard to the monomer composition (Fig. 3d), the 3HB proportion decreased, except for a slight increase before 12 h; the 3HV proportion increased in the feast phase and was then maintained at 23–26 mmol C% in the famine phase; the 3H2MV proportion gradually increased throughout. The composition of the PHA polymer at 24 h was expressed as 3HB, 3HV, and 3H2MV at 68.4, 23.7, and 7.9 mmol C%, respectively.

### Microbial community analysis of the enriched culture

**Figure 4:**
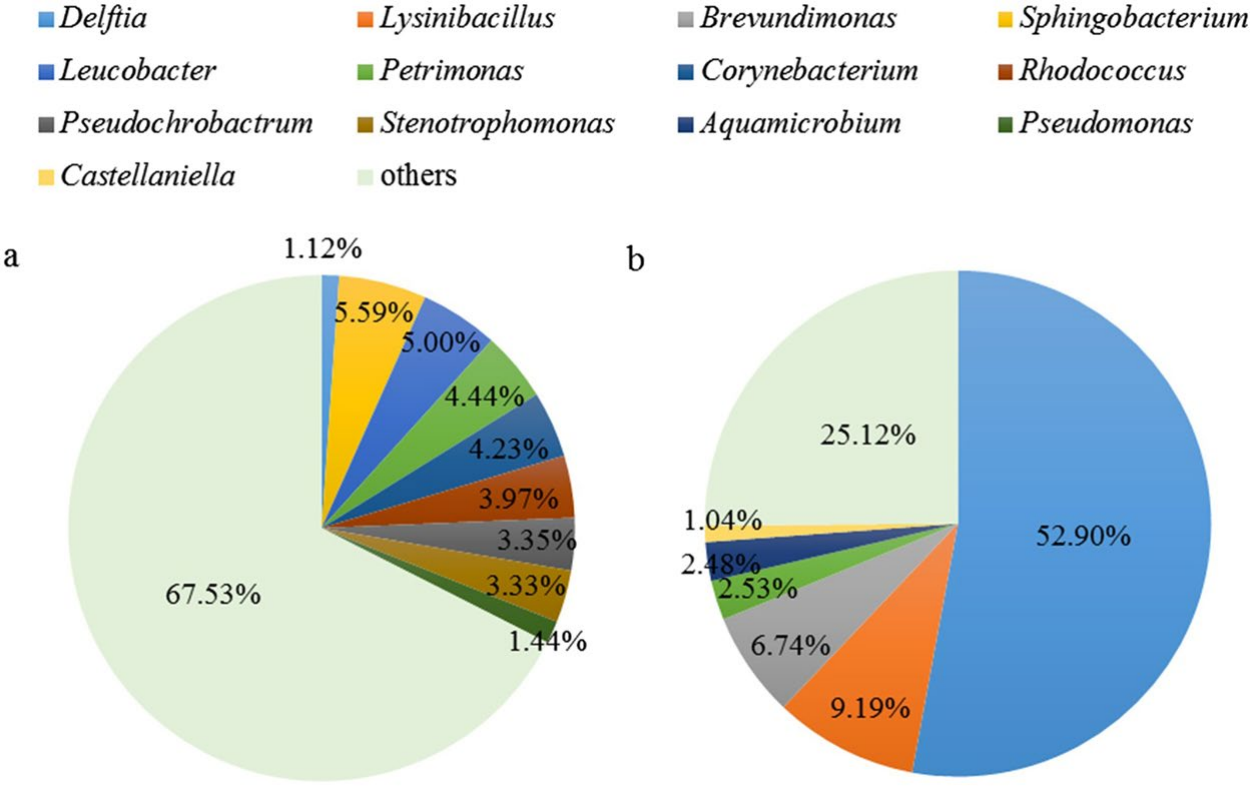
The relative abundance levels of genera in the microbial communities of the original activated sludge (**a**) and the enriched MMC (**b**). The genera with abundance less than 1% are classified into ‘others’.

The microbial communities of the original activated sludge and the enriched MMC were analyzed by high-throughput sequencing, and the optimized sequences were classified into different phylogenetic taxa. The results showed that for the enriched MMC at the phylum level, the bacteria belonging to Proteobacteria (79.80%), Firmicutes (14.41%), and Bacteroidetes (3.93%) were the three predominant groups, followed by those related to Actinobacteria (0.52%), Planctomycetes (0.38%) and Cyanobacteria (0.25%). According to the analysis at the genus level (Fig. 4), in the original sludge the sequences with more than 1% abundance were affiliated with *Sphingobacterium*, *Leucobacter*, *Petrimonas*, *Corynebacterium*, *Rhodococcus*, *Pseudochrobactrum*, *Stenotrophomonas*, *Pseudomonas* and *Delftia*. However, there was not a genus with the distinct advantage, and most genera (67.53%) had the respective abundance less than 1%, suggesting that the microbial diversity of the raw sludge was very high. While in the enriched MMC, the sequences mainly belonged to *Delftia*, *Lysinibacillus*, *Brevundimonas*, *Petrimonas*, *Aquamicrobium* and *Castellaniella*. Apparently, the genus *Delftia* was highly enriched (from 1.12% to 52.90%), and became the most important functional group in the enriched MMC. *Delftia* is a well-known PHA-producing group^34^, and the related bacteria in this genus have a strong adaptability to complex environments, such as municipal sludge and dirty water, with a wide substrate range from slaughterhouse waste to palm oil mill effluent^12^. Other genera in the MMC, such as *Lysinibacillus*, *Brevundimonas*, and *Castellaniella*, were also typical PHA producers^35^.

## Discussion

In the present study, efficient PHA production was achieved using the valerate-dominant sludge hydrolysate in an enriched MMC. The hydrolysate exhibited complex compositions, with high levels of non-VFAs and nutrients, differing substantially from manually synthesized feedstocks. The results revealed that valerate-dominant sludge hydrolysate was suitable to produce high-quality PHA containing 3HV and 3H2MV monomers. After being assimilated into the cell, valerate is converted to acetyl-CoA and propionyl-CoA, which are the precursors for 3HV and 3H2MV production^21^. The proportion of these two monomers in the produced PHA reached up to 31.6 mmol C%, which represented the highest value to date from MMCs with fermented wastes^4, 36^.

The enriched MMC displayed a higher PHA-producing ability than those previously observed in feast-famine PHA studies with acetate-dominant complex feedstocks containing non-VFAs and nutrients. The previously reported PHA contents were lower than 30%, although in a separate accumulation stage adopting a multiple feeding strategy, for example, 4% in the enrichment stage and 25% in the accumulation stage reported for a high-pressure thermophilic fermented sludge hydrolysate^30^, and 20% in the enrichment stage and 29% in the accumulation stage reported for a leachate from municipal solid waste^24^. By contrast, in the present study, more than 40% of the PHA content was obtained in the enrichment stage. Furthermore, the maximum PHA and CDW concentrations were at higher levels than in previous results. The maximum PHA production (1.30 g/L, equivalent to 60.47 C mmol/L) exceeded those reported with fermented wastes, such as 0.28 g/L for fermented diary manure^2^, 0.71 g/L for fermented sludge^9^, and 0.75 g/L for fermented sugar molasses^36^. To the best of our knowledge, the maximum CDW (3.08 g/L) was the highest obtained thus far by MMCs^10, 37^, indeed at similar or higher levels than pure cultures^14^. These high PHA and CDW concentrations were highly conducive to PHA product recovery^38^.

There are two main reasons why the MMC possessed a strong PHA synthetic ability. First, the enrichment method with an increasing substrate concentration was performed. This method was able to preserve as many PHA-producing bacteria as possible and provide enough time for them to fully display their synthetic potential. By contrast, the method with a fixed high VFAs concentration might easily destroy some PHA-producing bacteria^39^. Second, as odd-carbon VFAs were generally considered more difficult to be used than even-carbon VFAs in microbial central metabolism^32^, the MMC adapted to a high-valerate environment would be more robust than the ordinary cultures enriched by acetate-dominant or butyrate-dominant hydrolysates. The valerate-preferred MMC could better confront complex components and resist the latent inhibition of non-VFAs and nutrients.

Based on the microbial analysis, the enriched MMC revealed particular characteristics of community composition, and *Delftia* was first observed to exceed 50% percentage in the PHA enrichment. This might be because *Delftia* had a close affinity with valerate. It was reported that the pure culture of *Delftia acidovorans* displayed rapid valerate uptake and formed PHBV containing up to 90 mol% 3HV^13^. Despite the fact that the microbial community was also related to the origin of activated sludge and the operating conditions of the reactor^11^, *Delftia* prevailed over other typical PHA-producing bacteria in the MMC, such as *Lysinibacillus* and *Brevundimonas* (Fig. 4), which were observed to be dominant groups in the PHA enrichments^35^. This behavior demonstrated that *Delftia* had better adaptability to valerate-dominant environment than did other PHA-producing bacteria.

New insights were gained concerning specific VFA uptake. For the first time, it was elucidated that the uptake rate of the odd-carbon VFA valerate was higher than that of the even-carbon VFAs acetate and butyrate, highlighting the flexibility of VFAs uptake. This provided evidence that the VFAs consumption sequence and extent were closely linked to the substrate for enrichment. The concentrations and percentages of different VFAs in the enrichment feedstock shaped the microbial composition of the enriched culture, as well as the PHA synthetic pathway^10^. Thereupon, the microbial composition directly affected the utilization of individual VFA, as different PHA-producing bacteria had specific VFA-uptake preferences^11^. When valerate exhibited absolute dominance compared to even-carbon VFAs in the enrichment substrate, the enriched MMC involved prevailing *Delftia*, reinforcing valerate consumption. Hence, the valerate-dominant hydrolysate played an important role in the selective utilization of VFAs by the MMC when used as the enriching substrate. In addition, the dynamic changes of the uptake rates regarding different VFAs (Fig. 1c) indicated that the VFAs utilization was concentration dependent.

The optimal range of combined N and P for PHA production was proposed as N/COD 2-15 mg/g and P/COD 0.5–3 mg/g, which could offer a consistent improvement of PHA productivity^26^. In this study, N/COD and P/COD in the valerate-dominant sludge hydrolysate were 57.66 and 5.56 mg/g, respectively. In addition to the original N and P in the hydrolysate, the decomposition of non-VFAs would generate more during the cycle. Therefore, the nutrients in the feedstock were much more than required. Although nutritional imbalance has been traditionally regarded as a prerequisite for PHA synthesis^1^, this may only be true when non-VFA substrates are used as precursors^40^, while VFA substrates may promote MMCs with a wider nutritional range. The PHA-producing bacteria sustained the competitiveness of PHA synthesis over biomass growth not only by limited nutrients, but also through their physiological features^41^, and some PHA-producing strains intrinsically had difficulties coping with nutrient starvation^42^. Additionally, adequate nutrients could help balance and stabilize growth and PHA synthesis during long-term operation^27^. However, when the amounts of the nutrients exceeded a certain limit (in this study, N/COD of 150 mg/g and P/COD of 15 mg/g), the PHA production was distinctly impaired, possibly due to the redundant biomass growth over PHA synthesis and the disturbance of microbial metabolism. Further research is necessary to investigate the limit value of N and P inhibiting PHA production with the valerate-dominant feedstock.

It was previously considered that VFAs favored PHA-producing species, while non-VFAs mostly gave rise to populations that did not store PHA^10^, and the fraction of non-storing biomass was proportional to the fraction of non-VFA COD in the substrate^4^. Accordingly, the non-VFAs in the feedstock were also related to the overall PHA production efficiency. Comparing the two main non-VFAs, the proteins consumption rates were always much higher than those of carbohydrates, more than 10 times higher before 18 h and 5 times higher from 18 h to 24 h (Fig. 1c). Jia *et al*. also observed that bovine serum albumin (BSA, protein) was consumed much more than glucose (carbohydrate) when they were equivalently served for MMC PHA production^9^. As a whole, the average ratio of VFAs to non-VFAs -q_tVFA_/-q_tnon-VFA_ was 13 in the feast phase and 6.5 in the early famine phase (from 24 h to 48 h). As reported, keeping this ratio above 5 was vital to maintain the dominance of PHA producers over non-PHA producers in the reactor^25^. Besides, the enriched MMC preferred decomposing intracellular PHA for growth and maintenance to consuming the extracellular non-VFAs after the VFAs were used up. This condition was crucial for the MMC to maintain the regular feast-famine response during long-term enrichment, as PHA degradation in the famine phase signified adequate selective pressure^35^. The regular feast-famine response could help to gradually wipe out the non-PHA producers without cellular PHA reserve.

**Figure 5:**
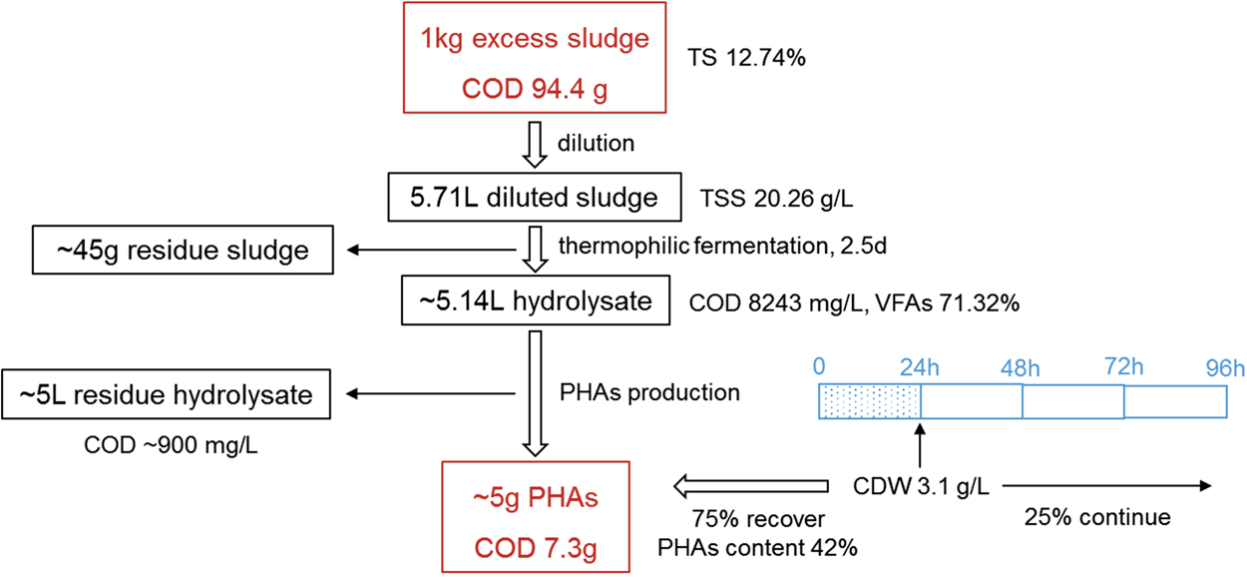
The overall process and efficiency of PHA production with valerate-dominant sludge hydrolysate in conversion of 1 kg excess sludge into PHA.

The yield of PHA over the two-stage process was estimated (Fig. 5), without the third-stage batch reactor which necessitated the additional time required for reactor preparation and operation. After 2.5 days of thermophilic anaerobic fermentation, approximately 900 mL of hydrolysate was collected from 1 of sludge mixture (90% volume recovery), and the solids reduction ratio was approximately 60% (40% left in the residue sludge). For the ADF cycle to recover the PHA product, the hydraulic retention time (HRT) was 4 d, and the biomass retention time (BRT) was 16 d. At 24 h, 75% of the microbial consortia (approximately 2.3 g/L) was collected for PHA recovery, and the other 25% (approximately 0.8 g/L) was left in the reactor throughout the famine phase. At the cycle end (96 h), the CDW decreased to approximately 0.35 g/L, and all of this biomass was passed to the next new cycle. This operation mode was tested at the laboratory scale (5 L) for more than two months (more than 20 cycles), and the reactor worked stably and efficiently. As shown in Fig. 5, 1 kg of excess sludge was estimated to result in 5 g of PHA product, that is, 94.4 g COD of sludge to 7.3 g COD of PHA, leading to a COD conversion efficiency of 7.73%. In a previous study, the potential of using a propionate-dominant hydrolysate to produce PHA was 1 kg of soluble COD in paper mill wastewater to 0.11 kg of PHA (approximately 0.16 kg COD of PHA)^19^, leading to an approximate COD conversion efficiency of 16%. However, most of the available substrates in the paper mill wastewater were soluble and much easier to be converted than the insoluble organics in sludge during anaerobic fermentation, also the fermented paper mill wastewater contained more VFAs in the total SCOD than the sludge fermented hydrolysate. Thus, the efficiency of the valerate-dominant sludge hydrolysate in the MMC PHA production was relatively lower.

The components of fermented streams can regulate the yield and composition of the PHA produced; then, the efficiency of the production process and the properties of the PHA product can be ameliorated from the initial anaerobic fermentation stage. However, the complexity and instability of fermented wastes make it difficult to grasp the mechanism of MMC PHA production, which limits the hydrolysate application. Consequently, it is essential to identify the specific mechanism with different hydrolysates, including the valerate-dominant sludge hydrolysate with non-VFAs and nutrients. Although a higher PHA content and concentration were obtained with complex feedstock in this study, ongoing research should be aimed at further optimizing the productivity and polymer properties, as well as improving anaerobic fermentation stage, for example, increasing the acidification degree and restricting nutrient release.

## Methods

### Sludge acidogenic fermentation

The excess sludge used for acidogenic fermentation was dewatered sludge collected from a municipal wastewater treatment plant in Beijing, China. The total solids (TS) content of the dewatered sludge was 12.74 ± 0.11% (w/w), and the volatile solids (VS)/TS ratio was 67.47 ± 0.20% (w/w). The dewatered sludge was diluted by deionized water, with 175 g of sludge to a total volume of 1 L. The diluted sludge was characterized as follows: pH = 6.8 ± 0.2, total suspended solids (TSS) = 20.26 ± 1.01 g/L, volatile suspended solids (VSS) = 13.98 ± 0.73 g/L, total chemical oxygen demand (TCOD) = 16539 ± 343 mg/L, soluble chemical oxygen demand (SCOD) = 296 ± 15 mg/L, total protein = 8287 ± 540 mg COD/L, total carbohydrate = 3668 ± 133 mg COD/L, and total lipid = 160 ± 8 mg COD/L. This diluted sludge was anaerobically fermented under thermophilic conditions (55 °C) in triplicate batch reactors with a 2 L working volume for 5 days, as described previously^17^.

To obtain the hydrolysate with a high VFAs concentration and the desired VFAs composition, the fermented mixture was withdrawn at indicated intervals to measure the SCOD and VFAs. The mixture was centrifuged at 10,000 rpm, 4 °C for 10 min, and the supernatant was so-called sludge thermophilic fermented hydrolysate. After analysis, the optimal time was determined to collect the hydrolysate and analyze it for soluble proteins, soluble carbohydrates, NH_4_-N and PO_3_-P. The collected fermented hydrolysate from different batches was mixed together and stored at 4 °C before use (no more than 72 h).

### Enrichment of the PHA-producing mixed culture

The activated sludge used to enrich the PHA-producing MMC was collected from the aeration tank of a municipal wastewater treatment plant in Beijing, China. The VSS was 9.36 ± 0.86 g/L, with an original PHA content of 1.17 ± 0.09% (w/w). The enrichment SBR with a 500 mL working volume was originally inoculated with 50 mL of activated sludge and 450 mL of diluted thermophilic fermented hydrolysate with an original SCOD of 500 mg/L. The SBR was situated in a shaker at 170 rpm, 30 ± 1 °C. The pH of the reactor was monitored but not controlled during reactor running. All the operations were conducted without sterilization. The length of the feast phase for each cycle was determined based on the SCOD and VFAs consumption, which were well correlated with the dissolved oxygen (DO) change^24^. The total cycle length (also HRT) was 4 times that of the feast phase, namely, a feast-to-famine ratio (F/F) of 1:3.

Several subsequent cycles were conducted in the same way as the first cycle until the reactor performances were stable. Then, the original SCOD of the substrate (diluted hydrolysate) was gradually increased to 1000 mg/L, 2000 mg/L, 3000 mg/L, 4000 mg/L, 6000 mg/L and, finally, an undiluted concentration. The hydrolysate was diluted by deionized water. For each concentration, the steady state was judged by examining the variations in the maximum PHA content and PHA concentration, with no more than a 5% standard deviation for the last three cycles. For cycles 1 to 3, at the cycle end after 10 min settlement, 250 mL of supernatant was withdrawn and centrifuged at 10,000 rpm and 4 °C for 5 min to obtain the bacterial consortium. For all the subsequent cycles, at the cycle end all 500 mL of media was withdrawn and centrifuged. At the beginning of each new cycle, 500 mL of fresh hydrolysate was supplied to evenly resuspend the settled bacterial consortium. The amount of the seeded biomass was controlled to achieve an initial optical density at 600 nm (OD_600_) of the media at 0.30 ± 0.03, and thus, the BRT for different cycles might change. The average of the values for three independent samples respectively from the last three cycles for each concentration represented one indicated value.

### Analytical methods

The parameters of TS, VS, TSS, VSS, SCOD, TCOD, NH_3_-N and PO_4_-P were analyzed according to standard methods^43^. The measurements of VFAs (including acetate, propionate, butyrate, and valerate), carbohydrate, protein, and lipid agreed with previous literature^17^. The OD_600_ of the mixed liquid in the PHA SBR was measured using a UV-1201 spectrophotometer (Shimadzu, Japan), to estimate the bacterial cell concentration. The samples from the SBR were centrifuged at 10,000 rpm and 4 °C for 10 min; the supernatant was analyzed for SCOD, soluble carbohydrates, soluble proteins, NH_3_-N and PO_4_-P after passing through a 0.45 µm filter, for VFAs through a 0.22 µm filter, while the sedimentary pellet was analyzed for cell dry weight (CDW) and PHA. The pellet was washed twice and stored at −80 °C overnight; then, the pre-frozen pellet was lyophilized for 48 h in a freeze dryer (Alpha 1-2LD plus, Christ, Germany). PHA extraction was performed according to the method of Jia *et al*.^9^. The monomers composition was measured using NMR spectroscopy (Ascend Aeon 900, Bruker, Switzerland). The concentration of each monomer was analyzed using gas chromatography (GC-2014, Shimadzu, Japan). The calibration curve of 3HB and that of 3HV were obtained by injecting the standard Poly(3-hydroxybutyric acid-co-3-hydroxyvaleric acid) (Sigma Aldrich, USA, CAS: 80181-31-3), while 3H2MV calibration curve was obtained by injecting its isomeride 2-Hydroxyhexanoic acid (Sigma Aldrich, USA, CAS: 6064-63-7).

The molecular weight and thermal properties of the produced PHA were characterized. The lyophilized microbial cells were processed via a CH_2_Cl_2_ solvent (10% (w/v), 50 °C, 8 h). PHA were precipitated in ice-cold methanol, air dried overnight and stored at 4 °C in the dark^38^. The weight-average molecular weight (*M*_*w*_) and number-average molecular weight (*M*_*n*_) were determined by gel permeation chromatography (GPC) relative to a polystyrene standard at 35 °C, using a 150 C GPC analysis system (Waters, USA). A sample concentration of 1.0 mg/mL was applied, and CHCl_3_ was used as the eluent at a flow rate of 1.0 mL/min^14^. For the analysis of thermal properties (including the melting point (*T*_*m*_) and the glass transition temperature (*T*_*g*_)), Q100 differential scanning calorimetry (DSC) (TA Instruments, USA) coupled with a liquid nitrogen cooling system was adopted in the temperature range of −40 to 200 °C at heating and cooling rates of 20 °C/min under a nitrogen atmosphere^44^.

### Microbial characterization

The biomass of the original activated sludge and the enriched MMC was collected for community analysis. To collect the MMC biomass, three independent samples respectively in the last three cycles for the final concentration were withdrawn at the end of the famine phase. The samples (original sludge and enriched MMC) were centrifuged at 10,000 rpm and 4 °C for 10 min, and equal amounts of the three settled biomass were pooled together for DNA extraction according to the published methods^3^. The extracted DNA was quantified using a NanoDrop 2000 Spectrophotometer (Thermo Fisher Scientific, USA). Illumina MiSeq high-throughput sequencing was implemented for microbial community analysis (CnKingBio, Beijing, China). Genomic DNA was amplified by targeting the V3 region of the bacterial 16 S rRNA gene. The raw sequences of original sludge and enriched MMC were deposited into the NCBI Sequence Read Archive (SRA) database respectively under the accession number SRR5631186 and SRR1948081. Computational analysis was performed in accordance with previously described methods^17^.

### Calculations

DA (%) was used to evaluate the acidogenic potential of the sludge and is expressed in Eq. 1 ^31^:

1 $$DA( \% )=\frac{{[VFA]}_{produced}-{[VFA]}_{initial}}{{[TCOD]}_{initial}-{[VFA]}_{initial}}$$

The VFAs and non-VFAs uptake rates (-q_VFA_ or -q_non-VFA_, mg COD /(L·h)) were calculated by dividing the amount of VFAs or non-VFAs consumed by the time elapsed for a specified period. PHA (mg) was the sum of the measured monomers (3HB, 3HV and 3H2MV). The PHA content (%) was expressed on a mass basis as mg PHA/mg CDW. The CDW included the active biomass (X) and PHA, and consequently, X (mg) was estimated as CDW ‒ PHA. The PHA monomer production rates (q_3HB_, q_3HV_ or q_3H2MV_, mg COD/(L·h)) were calculated by dividing the amount of PHA monomers produced by the time elapsed for a specified period. The growth rate q_X_ (mg COD/(L·h)) was calculated in a similar manner as PHA. The PHA productivity (Y_PHA/X_, mg COD/mg COD) was calculated based on the amount of PHA formed per biomass, and the accessibility of the VFAs (Y_VFA/X_, mg COD/mg COD) was calculated by dividing the amount of VFAs consumed by the biomass involved. COD equivalents were calculated according to conversion ratios of ×1.42 mg COD/mg, 3HB 1.38 mg COD/mg, 3HV 1.63 mg COD/mg, and 3H2MV 1.82 mg COD/mg.

## Electronic supplementary material

Supplementary Information
