# Supplementary material for: Overall process of using a valerate-dominant sludge hydrolysate to produce high-quality polyhydroxyalkanoates (PHA) in a mixed culture
Source: Sci Rep. 2017 Jul 31;7:6939. doi: 10.1038/s41598-017-07154-3 (PMC5537348; doi:10.1038/s41598-017-07154-3)
Supplement: Supplementary file 1 — Supplementary Information [file 41598_2017_7154_MOESM1_ESM.pdf]

Supplementary Information

Overall process of using a valerate-dominant sludge hydrolysate to produce high-quality polyhydroxyalkanoates (PHA) in a mixed culture

Jiuxiao Hao, Xiujin Wang, Hui Wang*

State Key Joint Laboratory of Environment Simulation and Pollution Control, School of Environment, Tsinghua University, Beijing 100084, China

*Corresponding author. E-mail: wanghui@tsinghua.edu.cn

Figures

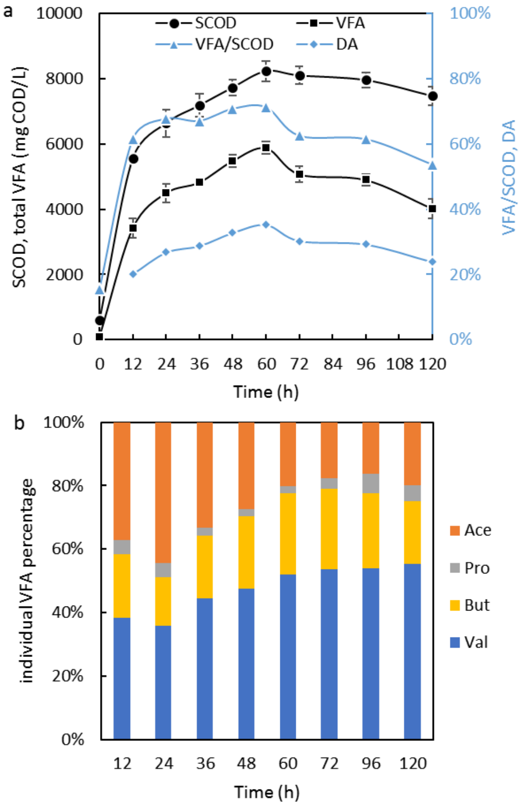

Figure S1. SCOD, total VFAs production (a) and the composition of individual VFA (b) during 5-day sludge thermophilic anaerobic fermentation. Ace: acetate; Pro: propionate; But: butyrate; Val: valerate. (bars = S.D., n = 3)

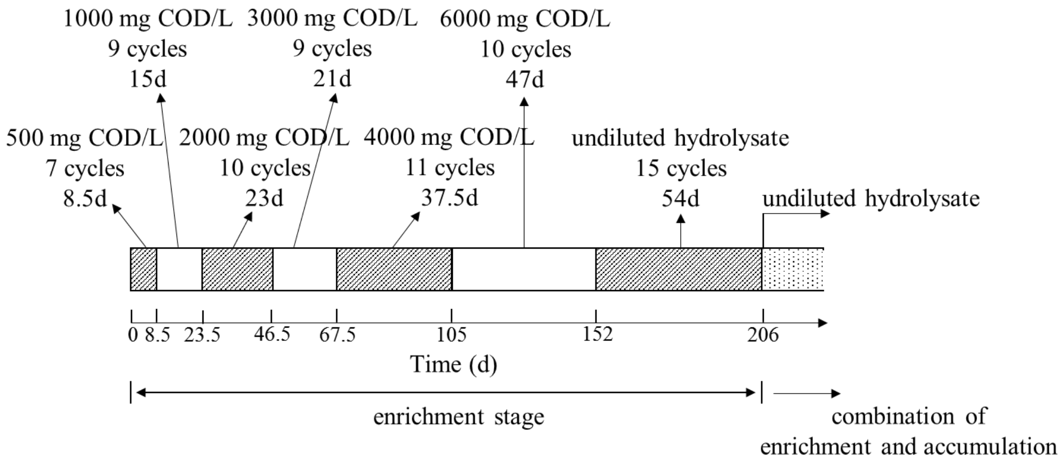

Figure S2. The whole process and timeline of enriching PHA-producing MMC fed by valerate-dominant sludge hydrolysate with increasing initial concentrations. After day 206, the PHAs product could be recovered from each cycle at the end of feast phase.

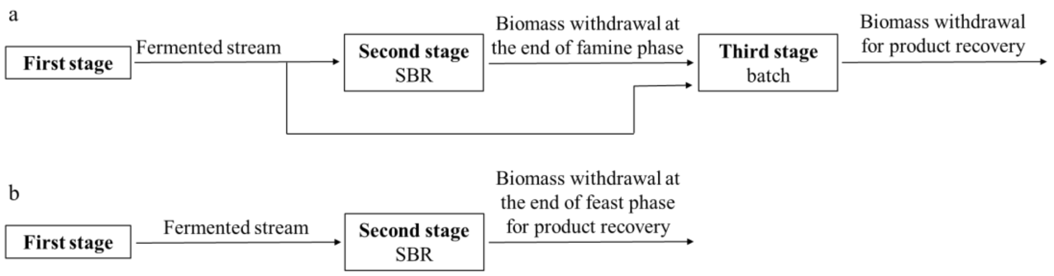

Figure S3. Comparison of conventional three-stage process (a) and two-stage process in this study (b) for PHAs production.
